# Constraining the sub-arc, parental magma composition for the giant Altiplano-Puna Volcanic Complex, northern Chile

**DOI:** 10.1038/s41598-020-63454-1

**Published:** 2020-04-22

**Authors:** Osvaldo González-Maurel, Frances M. Deegan, Petrus le Roux, Chris Harris, Valentin R. Troll, Benigno Godoy

**Affiliations:** 1 Department of Geological Sciences, University of Cape Town, Rondebosch, 7700 South Africa; 2 Departamento de Ciencias Geológicas, Universidad Católica del Norte, Avenida Angamos 0610, Antofagasta, Chile; 3 Department of Earth Sciences, Natural Resources and Sustainable Development (NRHU), Uppsala University, SE-75236 Uppsala, Sweden; 4 Instituto de Estudios Ambientales y Recursos Naturales (i-UNAT), Departamento de Física (Geología), Universidad de Las Palmas de Gran Canaria, Las Palmas de Gran Canaria, Spain; 5 Centro de Excelencia en Geotermia de los Andes (CEGA) y Departamento de Geología, Facultad de Ciencias Físicas y Matemáticas, Universidad de Chile, Plaza Ercilla 803, Santiago, Chile

**Keywords:** Geochemistry, Petrology, Volcanology

## Abstract

The Andean continental arc is built upon the thickest crust on Earth, whose eruption products reflect varying degrees of crustal assimilation. In order to robustly model magma evolution and assimilation at subduction zones such as the Andes, the compositions of parental magmas feeding crustal magma reservoirs need to be defined. Here we present new olivine and clinopyroxene oxygen isotope data from rare mafic volcanic rocks erupted at the margins of the giant Altiplano-Puna Magma Body (APMB) of the Altiplano-Puna Volcanic Complex, Central Andes. Existing olivine and pyroxene δ^18^O values for the Central Andes are highly variable and potentially not representative of sub-arc parental compositions. However, new olivine (n = 6) and clinopyroxene (n = 12) δ^18^O values of six Central Andean volcanoes presented here display a narrow range, with averages at 6.0‰ ± 0.2 (2σ S.D.) and 6.7‰ ± 0.3 (2σ S.D.), consistent with a common history for the investigated minerals. These data allow us to estimate the δ^18^O values of sub-arc, parental melts to ca. 7.0‰ ± 0.2 (2σ S.D.). Parental melts feeding the APMB and associated volcanic centres are postulated to form in the felsic continental crust following assimilation of up to 28% high-δ^18^O basement rocks by mantle-derived magmas.

## Introduction

**Figure 1:**
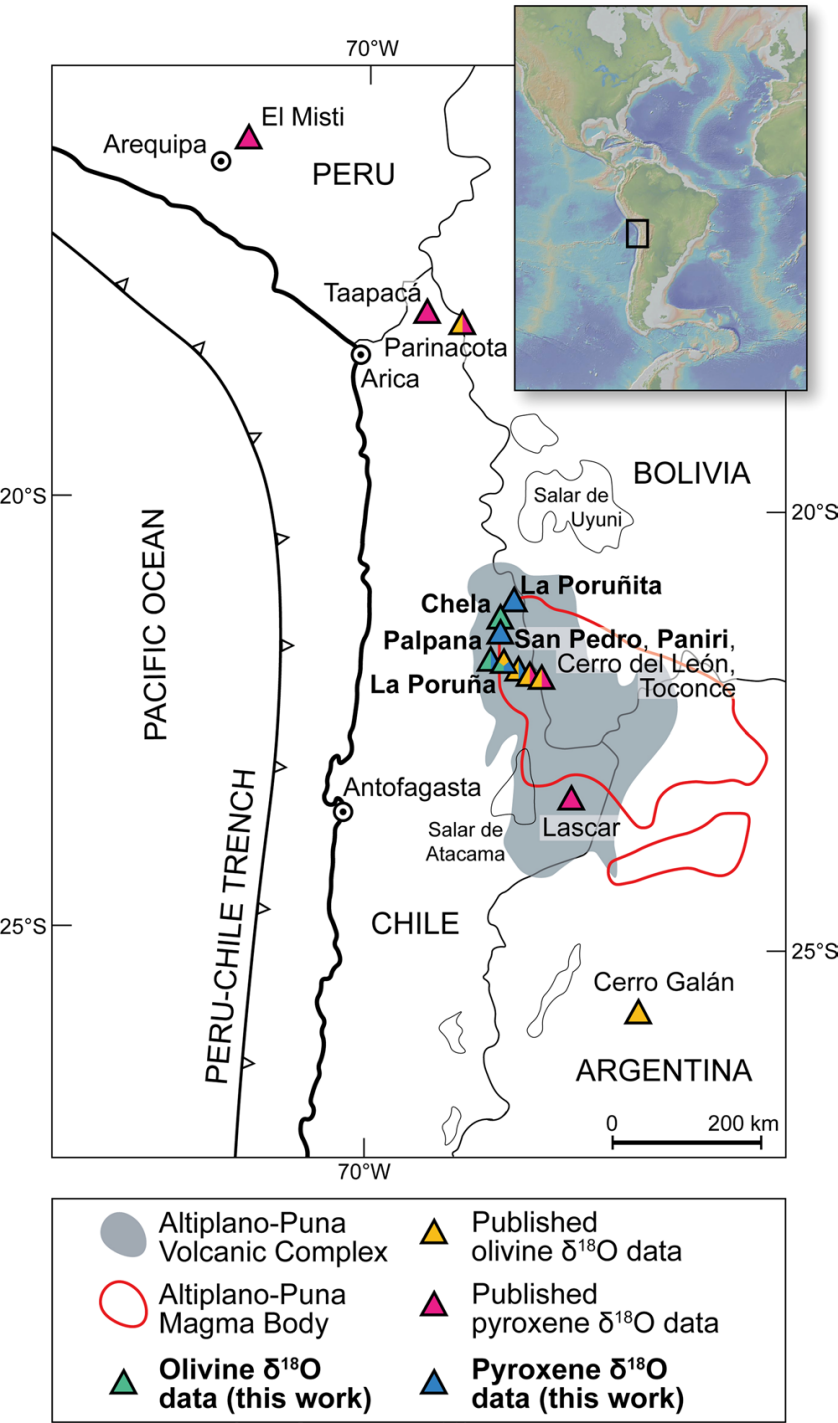
Study area. Map of the Central Andes showing the location of the volcanoes (in bold) included in this study and volcanoes with available δ^18^O values for olivine and pyroxene. The distribution of the Altiplano-Puna Volcanic Complex and the surface projection of Altiplano-Puna Magma Body are based on Zandt *et al*.^19^. Inset map created using “GeoMapApp” (www.geomapapp.org)^37^.

Eruption products of frontal arc volcanoes usually exhibit heterogeneous chemical and isotopic compositions because parental magmas are compositionally modified by incorporation of continental crust either at their source via subducted sedimentary material or by crustal contamination during subsequent ascent through the crust (e.g.^1^). A classic example of a volcanic arc, with near ubiquitous geochemical features of continental crust in its erupted products, is that of the Central Andes, which is associated with the thickest crust on Earth (70–74 km, ref. ^2^). The great thickness and compositional heterogeneity of the crust through which magmas must pass *en route* to the surface increases the likelihood for primitive magma compositions to be modified by crustal overprinting, yet knowledge of the primitive end-member is required in order to make robust models of subduction-related element fluxes. Oxygen isotopes allow robust modelling of crustal recycling in subduction zones as i) they undergo minimal fractionation at mantle temperatures, ii) there is a strong contrast between the δ^18^O values of mantle-derived magma and crustal rocks, and iii) the end-members involved have very similar O contents (e.g.^3–5^). In order to utilise oxygen isotopes to assess the magnitude of crustal material assimilated by evolved magmas along the Central Andean arc, it is necessary to know the δ^18^O value of the parent magma, which itself may be compositionally modified by assimilation in the deep crust. However, the oxygen isotope compositions of mafic magmas in the Central Andes are not well constrained and the existing δ^18^O values, including those obtained on olivine and pyroxene phenocrysts (Fig. 1), are highly variable (Supplementary Table S1). Existing oxygen isotope data obtained by conventional and laser fluorination analysis of olivine^6–8^ and pyroxene^6,8–10^ from Central Andean volcanoes have δ^18^O values ranging from 5.0 to 8.3‰ (average = 6.7‰, n = 19) and 5.5 to 8.7‰ (average = 6.2‰, n = 39), respectively (see Supplementary Tables S2 and S3). In contrast, typical mantle rocks show very limited variations in their δ^18^O values (e.g. olivine = 5.2 ± 0.3‰; clinopyroxene = 5.6 ± 0.4‰; ref. ^11^). The spread in the Central Andean literature data suggests that the oxygen isotope ratios of some of these lavas were modified by various processes post-dating the formation of the parental melt (e.g. extensive fractionation, late-stage assimilation, mixing of isotopically diverse magmas, or alteration). The challenge, therefore, is to constrain the parental melt value, before extensive fractionation or late-stage assimilation has taken place. We aim to achieve this goal by analysing the δ^18^O values of single minerals from rare, weakly differentiated lavas with relatively low silica contents (SiO_2_ = 54.6 to 57.2 wt%^12^) and relatively low Sr and high Nd isotope ratios (^87^Sr/^86^Sr = 0.70554 to 0.70669; ^143^Nd/^144^Nd = 0.51234 to 0.51251; ref. ^12^). The samples selected for this study are from six individual volcanoes (La Poruña, San Pedro, Paniri, La Poruñita, Palpana and Chela), which were active at different times and are all located around the western margin of the Altiplano-Puna Magma Body (APMB) melt anomaly in the Altiplano-Puna Volcanic Complex (Fig. 1). Based on their radiogenic isotope compositions, our samples have experienced limited degrees of crustal modification (e.g. assimilation) and are therefore ideally suited to obtaining the parental δ^18^O values locked in early-formed crystals. The crystal-focused approach we employ here offers critical new insights that have until now been under-explored due to the limitations inherent in whole-rock geochemical approaches (e.g. the susceptibility of whole-rock samples to secondary alteration and the fact that the δ^18^O values of whole-rock samples represent averages of the various phases that constitute the sample, cf.^1^). Our results therefore contribute to filling the gaps in our knowledge of subduction related parental magma compositions feeding the largest continental magma system on Earth.

### Study area and sample selection

Common volcanic products in the Central Andes include stratovolcanoes and extensive ignimbrite deposits, but several monogenetic volcanoes of mafic character also exist^12,13^. Within the volcano-tectonic ignimbrite province of the Altiplano-Puna Volcanic Complex^14^, the location of mafic volcanism is largely confined to the borders of the partial melt anomaly termed the Altiplano-Puna Magma Body (APMB^12^; Fig. 1). The APMB is the largest known zone of partial melting in the continental crust throughout the world, with an estimated melt volume of 500,000 km^3^ and spanning a region of ca. 200 km in diameter^15,16^ (Fig. 1). Based on geophysical surveys, this anomaly, located in the upper crust, shows an increasing melt fraction from its margin (ca. 4 vol%) to its centre (up to 25 vol%) (e.g.^16–18^). In this region, volcanoes outside the limits of the APMB are composed of lava that is more primitive than the volcanoes situated directly above the APMB^12^.

The volcanoes included in this study comprise, in order of increasing eruption age, La Poruña, San Pedro, Paniri, La Poruñita, Palpana and Chela, all of which are situated within the Altiplano-Puna Volcanic Complex but peripheral to the proposed APMB reservoir^19^ (Fig. 1). In this region, the ascending parental basaltic-andesite magma is thought to have avoided significant contamination by evolved melts from the APMB as demonstrated by the lowest ^87^Sr/^86^Sr and highest ^143^Nd/^144^Nd being towards the borders of the large felsic body^12,20^. The studied volcanoes (Fig. 1), together with the other Pliocene to Quaternary andesitic-to-dacitic stratovolcanoes, dacitic domes and monogenetic cones, overlie Miocene rhyodacitic-to-rhyolitic ignimbrite sheets^21^.

La Poruña (21°53′S; 68°30′W) is a well-preserved 140 m high scoria cone 100 ka in age^22^ situated on the west flank of the 6000 m San Pedro stratovolcano complex (21°53′S; 68°24′W). La Poruña is composed of pyroclastic material and an extensive basaltic-andesite to andesite lava flow that extends up to 8 km to the south-west of the main vent, whereas San Pedro is a composite stratovolcano formed by two superimposed coalescent cones^21^. The entire La Poruña volcano represents a monogenetic, relatively small to medium volume and short-lived singular eruption, whose magmatic evolution has been described as a two-stage evolutionary process involving minor assimilation and fractionation, followed by selective assimilation during turbulent ascent^22^. In contrast, San Pedro is a >100 km^2^ andesitic-to-dacitic volcanic field, with a long-lived (from ca. 510 ka to present) but episodic eruptive centre, whose recent mafic activity (<160 ka) is genetically similar to La Poruña^22^. Paniri (22°03′S; 68°14′W) is a stratovolcano constructed during four separate stages between 1.4 Ma to 100 ka, whose most primitive activity is represented by isolated basaltic-andesite to andesite lava flows erupted at ca. 400 kyr ago^23^. La Poruñita (21°17′S; 68°15′W), situated in the northernmost part of the projected APMB, is a scoria cone ca. 600 ka in age of about 700 m in diameter^24^, similar in shape and composition to La Poruña^12^. Palpana (21°32′S; 68°31′W) is a conical stratovolcano built up of mafic andesite lava flows. The summit of the volcanic edifice has a crater morphology (dimensions 1.8 km by 1.3 km) that is truncated by the last-erupted dome^21^. Chela volcano (21°24′S; 68°30′W) is very similar in shape and composition to Palpana. The shape and relatively monotonous composition have been related to rapid construction of the volcanic edifices at ca. 4.1 Ma for Chela and ca. 3.8 Ma for Palpana, followed by restricted erosion and limited duration of magmatic differentiation^24^.

Olivine- and pyroxene-phyric lava and scoria are ubiquitous at La Poruña, San Pedro, Paniri, La Poruñita, Palpana and Chela and vary from basaltic-andesite to andesite in composition, with whole-rock elemental and Sr and Nd isotope compositions that range from e.g., SiO_2_ = 54.6 to 62.9 wt%, MgO = 1.6 to 6.1 wt%, Sr = 389 to 885 ppm, Cr = 5 to 625 ppm, ^87^Sr/^86^Sr = 0.705541(10) to 0.707656(10), and ^143^Nd/^144^Nd = 0.512337(12) to 0.512513(50) (see^12,22,23^). Recent work on these volcanoes utilised whole-rock elemental and Sr and Nd isotope data to construct an evolutionary model, in which limited magmatic differentiation occurred at mid-upper crustal levels^12,22^. Lavas of these selected volcanoes may thus represent the composition of parental magmas feeding volcanism within the Altiplano-Puna Volcanic Complex, as the magmas feeding these mafic eruptions largely escaped assimilation of APMB felsic melts during ascent^12^. In this study, we focussed on sample material containing suitable mafic mineral phases for single mineral oxygen isotope analysis.

## Results

### Petrography

**Figure 2:**
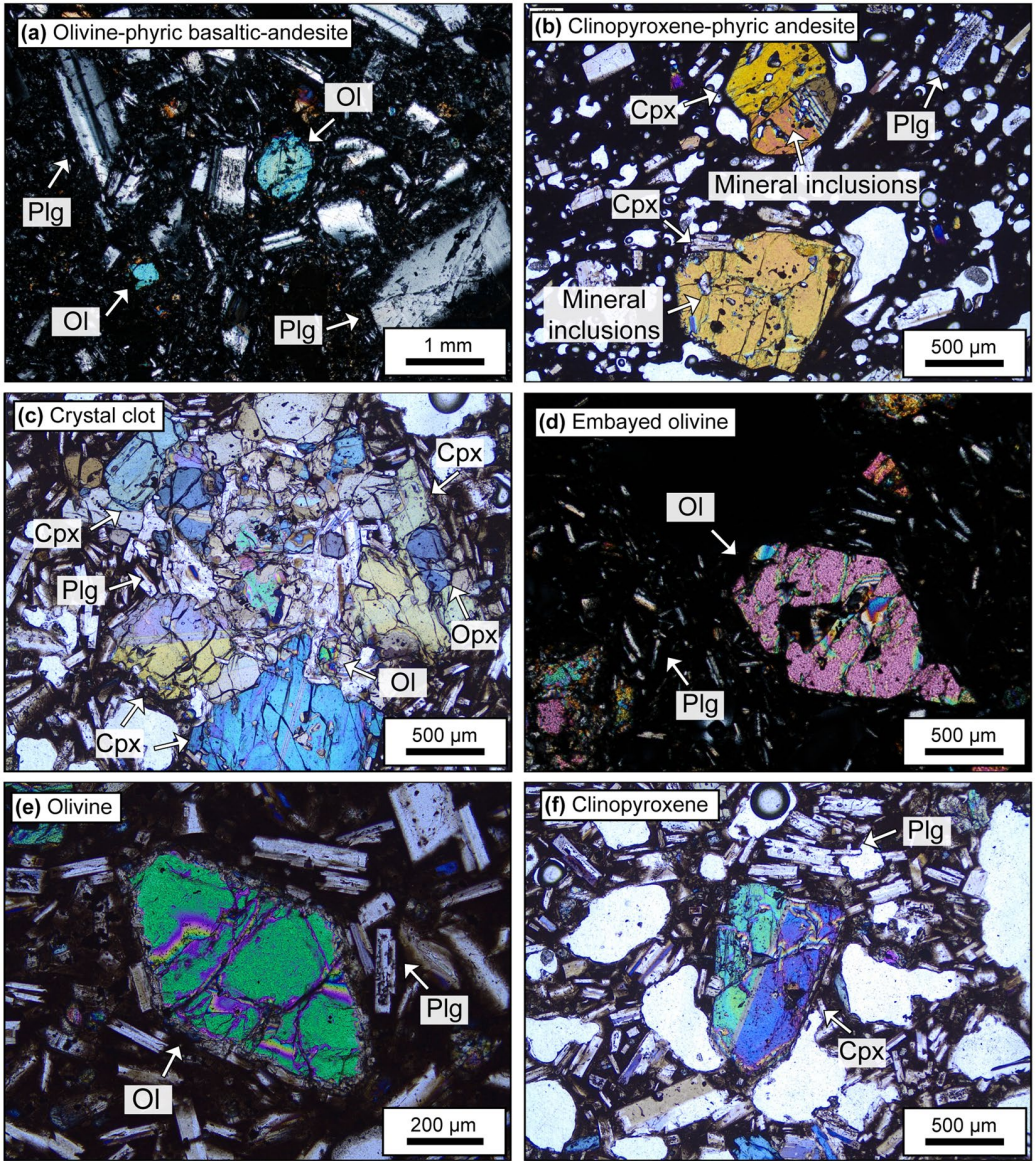
Selected photomicrographs under cross-polarized light of the studied samples. (**a**) CHE-03, fine-grained seriate textured plagioclase-olivine-phyric basaltic-andesite. (**b**) PAL-02, glassy fine-grained plagioclase-pyroxene-phyric andesite. (**c**) POR-06, crystal clot of clinopyroxene, plagioclase, orthopyroxene and olivine. (**d**) SPE-10, embayed olivine in a glassy olivine-pyroxene basaltic-andesite (**e**) POR-06, euhedral olivine crystal set in a microlite-rich groundmass of plagioclase and glass. Olivine crystals usually have fractures and mineral inclusions. (f) POR-06, subhedral clinopyroxene crystal. Clinopyroxene crystals generally contain melt or mineral inclusions, and visible twinning and zoning.

Basaltic-andesite lavas from La Poruña contain ca. 30 vol.% phenocrysts (plagioclase > olivine > clinopyroxene > orthopyroxene) and Fe-Ti oxides set in a microlite-rich groundmass of plagioclase and pyroxene and a small percentage of remaining glass. Olivine (up to 2.5 mm in size; ≤12 vol.%) textures include subhedral crystals, embayments and skeletal textures. Clinopyroxene is the most common pyroxene phase and occurs as euhedral to subhedral individual crystals (up to 2 mm; ≤10 vol.%) or as reaction rims on orthopyroxene phenocrysts. Besides occurring individually, clinopyroxene crystals occur as glomerocrysts with plagioclase, olivine and orthopyroxene (Fig. 2).

Basaltic-andesites from San Pedro are porphyritic (ca. 15 vol.% phenocrysts), glassy and fine-grained, with plagioclase > olivine > clinopyroxene > orthopyroxene as phenocrysts. Olivine (up to 2 mm; ≤10 vol%) appears frequently embayed, resorbed, or with reaction rims of pyroxene (Fig. 2). Clinopyroxene appears as euhedral to subhedral phenocrysts (up to 1.5 mm; ≤8 vol.%). Clinopyroxene phenocrysts occur individually or as crystal clots.

Basaltic-andesites from La Poruñita are vesicular and fine-grained, containing olivine > clinopyroxene = orthopyroxene as phenocrysts (≤15 vol.%). These phenocrysts are set in a hypocrystalline groundmass, which is made of microlites of plagioclase, interstitial glass, clinopyroxene and orthopyroxene. Olivine morphologies are varied, including subhedral and resorbed crystals (up to 0.5 mm in size; ≤7 vol.%). Most of these phenocrysts show embayments and oxide inclusions. Clinopyroxene appears as euhedral to subhedral individual phenocrysts (up to 1.5 mm; ≤4 vol.%).

Basaltic-andesites from Paniri are plagioclase-olivine-pyroxene-phyric lavas. These contain ca. 30 vol.% phenocrysts of plagioclase > olivine = clinopyroxene = orthopyroxene. Olivine and pyroxene frequently form glomerocrysts with plagioclase. Clinopyroxene (up to 1.5 mm in size; ≤8 vol.%) also appears individually as euhedral to subhedral crystals.

Andesites from Palpana contain ca. 30 vol.% phenocrysts of plagioclase > clinopyroxene > orthopyroxene and minor olivine set in a hypocrystalline groundmass of glass, plagioclase and pyroxene. Clinopyroxene (up to 2.5 mm; ≤15 vol.%) occurs as subhedral to euhedral tabular crystals (Fig. 2).

Basaltic-andesites from Chela are composed of ca. 25 vol.% phenocrysts (plagioclase > olivine» clinopyroxene > orthopyroxene) set in a glassy groundmass. Olivine generally appears as subhedral crystals in a seriate crystal size distribution as phenocryst and microlites (up to 2 mm; ≤10 vol.%; Fig. 2). Glomerocrysts (olivine-pyroxene) and pyroxene reaction rims on olivine are common. Orthopyroxene and clinopyroxene occur only rarely as glomerocrysts and microlites.

### Oxygen isotope data

**Figure 3:**
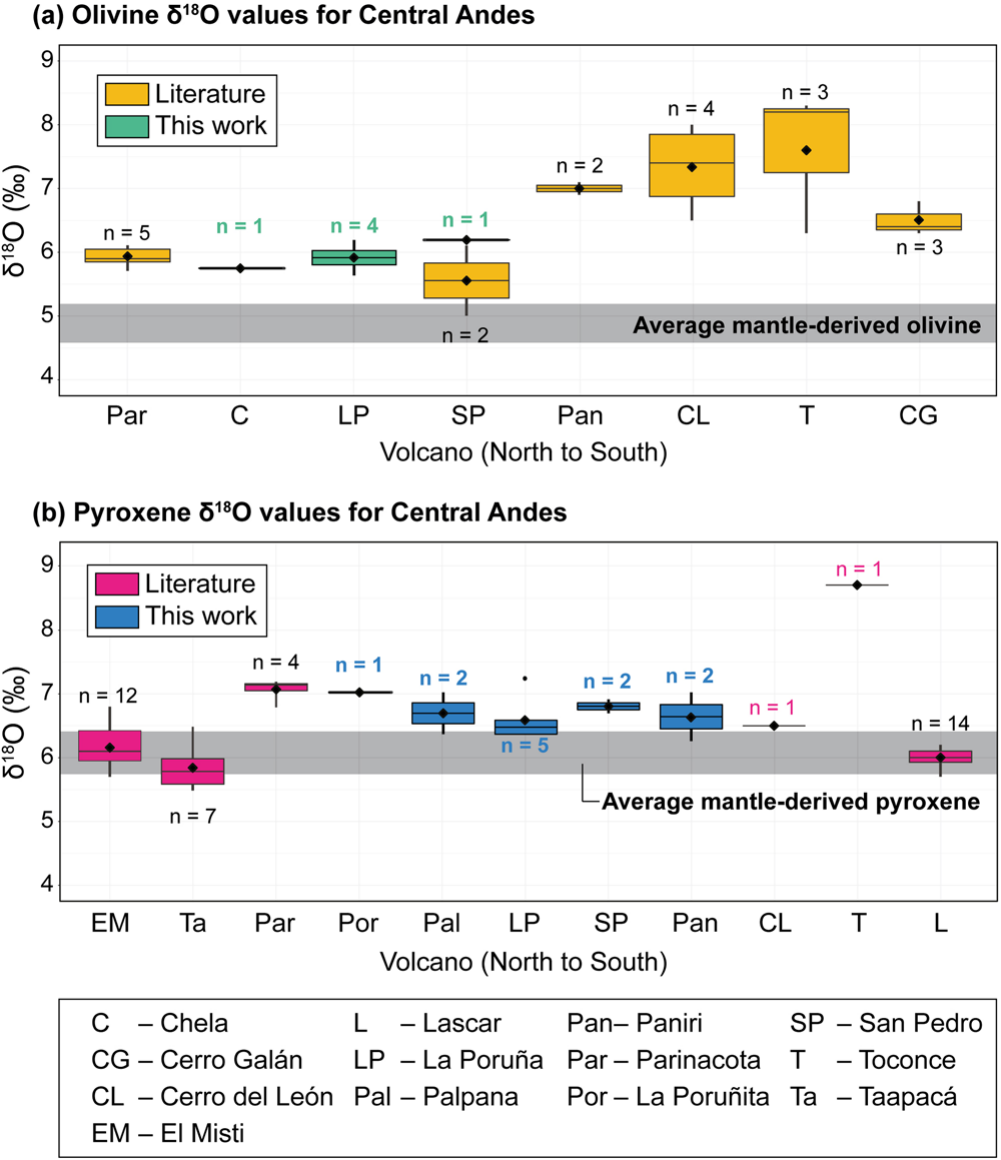
Oxygen isotope data for the Central Andes. (**a,b**) Variation in new (in bold) and literature δ^18^O values for olivine (**a**) and pyroxene (**b**) obtained by conventional (i.e. Parinacota^6^) and laser fluorination and displayed as box-and-whisker plots. Note that the new olivine and clinopyroxene data have higher average δ^18^O values for olivine and pyroxene than mantle-derived basalts (average = 4.8‰ ± 0.2, n = 104 for olivine and average = 6.1‰ ± 0.3, n = 16 for pyroxene^25^). Further details of published data are given in Supplementary Tables S2 and S3.

We determined the δ^18^O values of (i) olivine from lavas from three volcanoes whose erupted products contain large (≥2 mm) olivine phenocrysts (La Poruña, San Pedro, and Chela) and (ii) clinopyroxene from five volcanoes whose erupted products contain large (≥1.5 mm) pyroxene phenocrysts (La Poruña, San Pedro, Paniri, La Poruñita and Palpana) (Table 1). All minerals were individually selected under a binocular microscope before analysis and were visually free of inclusions or alteration. Laser Fluorination (LF, see Methods) analysis of olivine gave δ^18^O values of 5.7 to 6.2‰ for La Poruña (n = 4), 6.2‰ for San Pedro (n = 1), and 5.8‰ for Chela (n = 1). These values overlap the higher values obtained for olivine from mantle-derived basalts (cf. up to 6.3‰^25^; Fig. 3). Individual clinopyroxene crystals analysed by LF for La Poruña, San Pedro, La Poruñita and Palpana volcanoes gave average δ^18^O values of 6.4 to 7.2‰ (n = 5), 6.7 to 6.9‰ (n = 2), 7.0‰ (n = 1) and 6.3 to 7.0‰ (n = 2), respectively. The clinopyroxene crystals analysed here have higher δ^18^O values than mantle-derived pyroxene (cf. up to 6.5‰, after^25^; Fig. 3).

**Table 1:** Laser fluorination analyses of olivine and clinopyroxene crystals from selected mafic volcanic rocks erupted at the western margin of the Altiplano-Puna Volcanic Complex. Whole-rock geochemical and isotope composition from González-Maurel *et al*.^12^.

Volcano (North to South)	Sample	δ^18^O_ol_	δ^18^O_melt-ol_	δ^18^O_cpx_	δ^18^O_melt-cpx_	Whole-rock geochemistry and Sr and Nd isotopes					
						*SiO*_*2*_ *(wt%)*	*MgO (wt%)*	*Mg#*	*Sr (ppm)*	^*87*^*Sr/*^*86*^*Sr*	^*143*^*Nd/*^*144*^*Nd*
La Poruñita	PORU-01			7.0	7.7	55.49	4.28	52	484	0.706408(13)	0.512344(12)
Palpana	PAL-02			6.4	7.1	57.21	3.82	52	632	0.705730(11)	0.512426(11)
				7.0	7.7						
Chela	CHE-03	5.8	7.1			54.61	3.86	49	793	0.705541(10)	0.512513(50)
La Poruña	POR-05	5.7	7.0	6.4	7.1	56.28	4.09	57	452	0.706441(10)	0.512444(13)
				6.6	7.3						
	POR-06	5.9	7.2	6.4	7.1	55.61	6.08	61	514	0.706361(12)	0.512419(17)
				6.5	7.2						
	CH-AZU-010	6.0	7.3								
		6.2	7.5								
	POR-08			7.2	7.9	55.78	5.52	59	456	0.706679(12)	0.512385(14)
San Pedro	SPE-10	6.2	7.5	6.7	7.4	55.17	5.50	59	558	0.706672(11)	0.512374(14)
				6.9	7.6						
Paniri	PANI-05			6.3	7.0	56.04	4.09	53	599	0.706690(11)	0.512374(14)
				7.0	7.7						

The oxygen isotope data are reported in ‰ relative to the V-SMOW scale. See Methods for analytical details. *ol* olivine, *cpx* clinopyroxene. The estimations of melt δ^18^O values (i.e. δ^18^O_melt-ol_ and δ^18^O_melt-cpx_) are based on an equilibrium mineral-melt fractionation factor of 1.3‰ for olivine and 0.7‰ for clinopyroxene (see text for details; mineral-melt fractionations from Bindeman *et al*.^29^).

**Figure 4:**
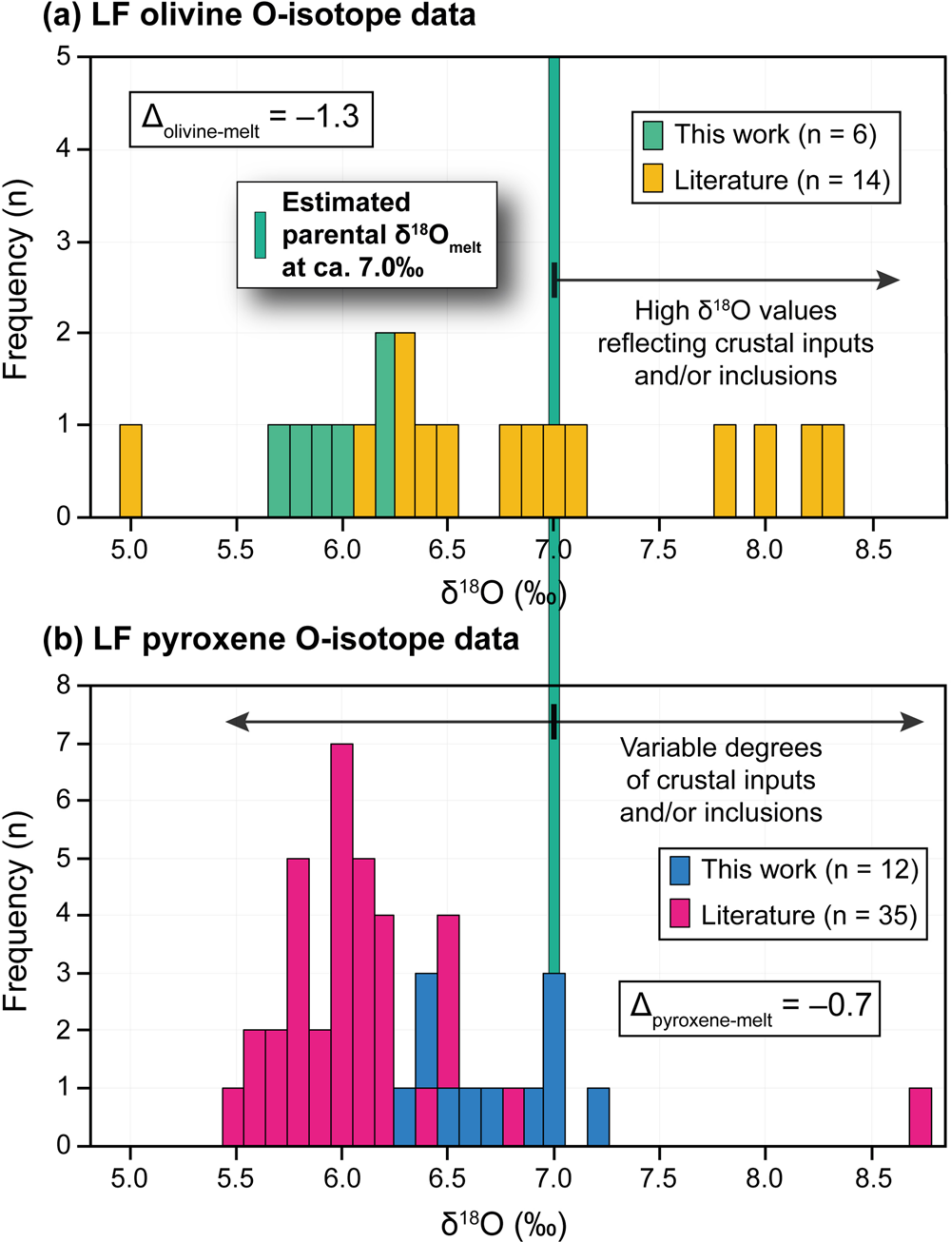
Frequency distribution of δ^18^O values of olivine (**a**) and pyroxene (**b**) for the new data in comparison to published data obtained by laser fluorination from the Central Andes. Note that new olivine data overlap with the lowest δ^18^O values reported previously for the Central Andes, whereas new clinopyroxene data overlap the higher end of the δ^18^O range. The equilibrium melt would have had a δ^18^O value of ca. 7.0‰, based on olivine-melt and clinopyroxene-melt fractionation factors of 1.3‰ and 0.7‰, respectively (see text; mineral-melt fractionations from Bindeman *et al*.^29^). Summary of previously published data are given in Supplementary Tables S2 and S3.

Our olivine and pyroxene δ^18^O values display substantially narrower ranges than the available data for the Central Andes (Fig. 3). Published olivine δ^18^O values^6–10^ tend to have either relatively high (>6.5‰) or mantle-like δ^18^O values. Notably, our olivine oxygen isotope data from La Poruña, San Pedro and Chela volcanoes have among the lowest δ^18^O values (δ^18^O = 5.7% to 6.2‰) with respect to all olivine data reported thus far for the Central Andes (cf. Parinacota^6^; Cerro Galán^7^; San Pedro^8^; Fig. 4). Our clinopyroxene data (δ^18^O = 6.3% to 7.2‰) overlap with the δ^18^O values previously obtained for pyroxene from the Central Andes (5.5‰ to 8.7‰^6,8–10^) and are at the higher end of the previously reported data range (excluding one exceptionally high value reported for Toconce volcano^8^; Fig. 3).

## Discussion

Available whole-rock geochemistry for the studied samples (Table 1) reveal that clinopyroxene-phyric (e.g. PAL-02) and olivine-pyroxene-phyric lavas (e.g. POR-06) have higher SiO_2_ contents than samples that only contain olivine as phenocrysts (e.g. CHE-03). It is thus possible that pyroxene crystallised at a higher crustal level than olivine and might record late-stage crustal assimilation (cf.^26–28^). We also note that arc lava pyroxenes frequently contain inclusions of plagioclase (which would have higher δ^18^O values) and/or oxides (lower δ^18^O values) (e.g. Fig. 2; see also Fig. 4 in Deegan *et al*.^26^). Because of the very dark appearance of pyroxene under the binocular microscope, it is both difficult to determine if inclusions are present and what they are. The wider variation of pyroxene δ^18^O values in this study, compared to olivine may, therefore, be due to either late-stage crustal assimilation or inclusions of various types in the analysed material.

It is possible to estimate the δ^18^O values of the equilibrium melt by using mineral-melt fractionation factors appropriate for basaltic-andesite (SiO_2_ average 55.8 wt% among our samples; Table 1). These are calculated to be Δ_olivine-melt_ = −1.3 and Δ_pyroxene-melt_ = −0.7, using the silica-based equations in Bindeman *et al*.^29^. Olivine with δ^18^O values of 5.7 to 6.2‰, therefore, crystallised from magma having a δ^18^O value of 7.0 to 7.5‰ (average = 7.3‰ ± 0.17, n = 6). Clinopyroxene with δ^18^O values between 6.3 and 7.2‰ similarly calculates to magma δ^18^O values of 7.0 to 7.9‰ (average = 7.4‰ ± 0.29, n = 12). These magma δ^18^O values are within error of each other but are up to 2.0‰ higher than the accepted values for normal mid-ocean ridge basalts (N-MORB) (δ^18^O = 5.4‰ to 5.8‰^30^) and MORB glass (δ^18^O = 5.4 to 5.8‰^31^). They are also higher than previously reported δ^18^O values from mantle-derived rocks in subduction zones elsewhere (e.g. δ^18^O ≤ 6.3‰^5,26,31,32^). Given that our samples have relatively high SiO_2_ contents and Mg numbers that range from 54.6 to 57.2 wt% and 49 to 61, respectively (Table 1), they are unlikely to represent primary or primitive mantle-derived magmas. Indeed, the O-isotope data presented here suggest assimilation of e.g. high-δ^18^O felsic continental crust resulting in an ^18^O-enriched parental magma.

**Figure 5:**
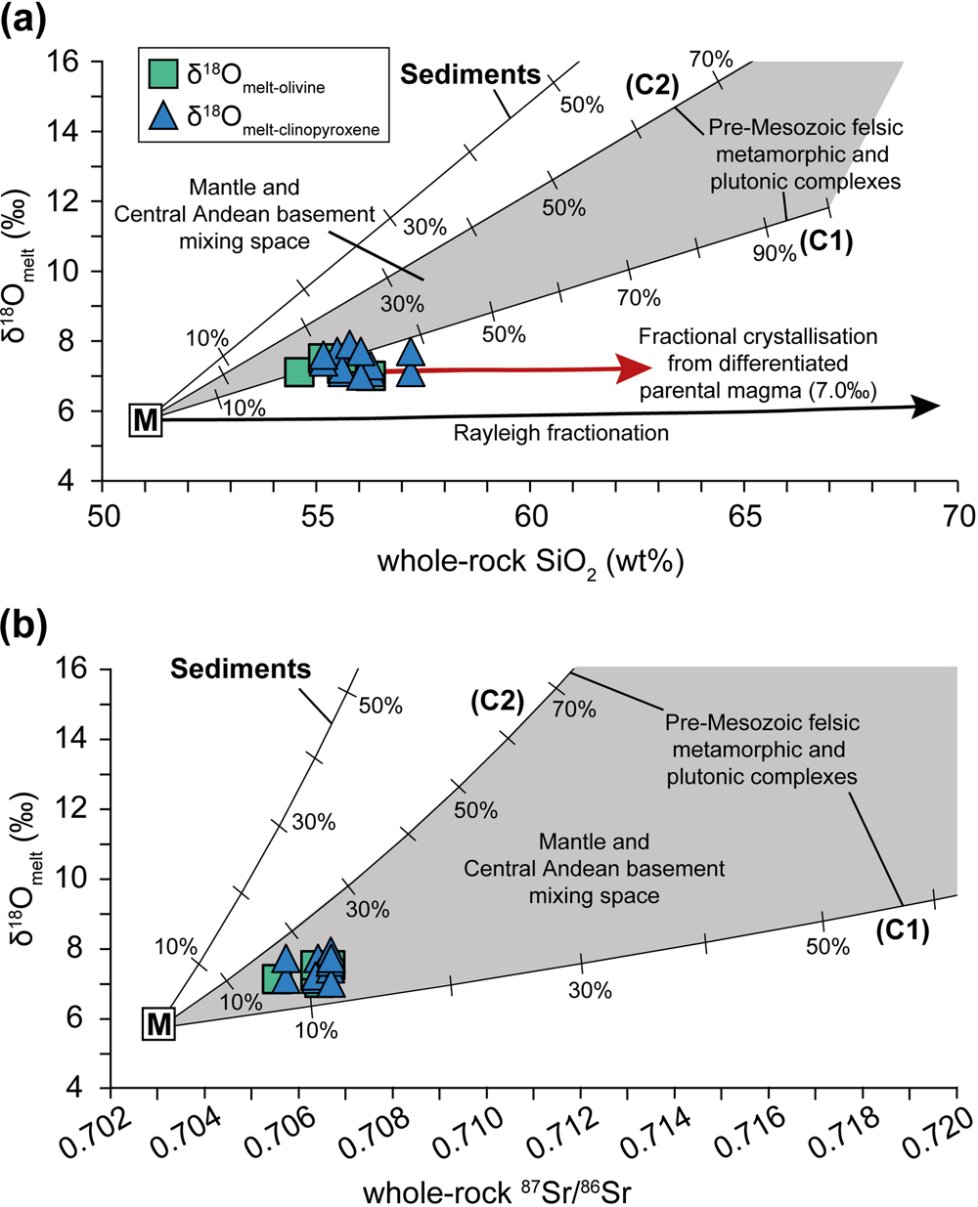
Binary mixing models of δ^18^O estimated melt values from analysed olivine and clinopyroxene in this work versus whole-rock (**a**) SiO_2_ and (**b**) ^87^Sr/^86^Sr ratios from González-Maurel *et al*.^12^. The classical Rayleigh fractionation trend illustrates the variation in δ^18^O values expected from closed-system fractional crystallisation. Curves *C1*, *C*2 and *Sediments* indicate possible types of local crustal contaminants or recycled components. The sub-arc, parental melt δ^18^O values of 7.0‰ ± 0.2 (2σ S.D.) are postulated to reflect mantle-derived magmas (M) assimilating significant amounts of high-δ^18^O continental crust (C1 and C2). Following crustal assimilation by M, parental magmas appear to undergo closed-system fractional crystallisation, i.e. increasing magma SiO_2_ at constant δ^18^O. Additional data sources are shown in Supplementary Table S6.

The high calculated melt δ^18^O values presented here cannot be explained by closed-system Rayleigh fractionation (see calculated curve in Fig. 5) as this would only increase primitive δ^18^O values by 0.2 to 0.3‰ (e.g.^29^). Pre-Mesozoic felsic metamorphic and plutonic complexes form the Central Andean basement of northern Chile at ca. 18°S to 25°S have δ^18^O values that range between 6.4‰ to 11.8‰^33^. If it is assumed that the mantle-derived magma had a δ^18^O value of 5.7‰ (e.g.^34^), a minimum of approximately 21% assimilation of local crust with a δ^18^O of 11.8‰ would be required to reach a magma value of 7.0‰, using simple mass balance calculations (X = [δ^18^O_final_ − δ^18^O_initial_]/[δ^18^O_assimilant_ − δ^18^O_initial_], where X is the amount of contamination as a fraction) and assuming equal oxygen content for all end-members. This estimated degree of assimilation agrees well with the estimates based on radiogenic isotope and trace element modelling using data from the same samples, which require about 12 to 28% assimilation (see Supplementary Information), in broad agreement with recent findings for the studied volcanoes (cf. ~13% to 23%^20^). Binary mixing modelling shows that our data are best explained by interaction between primitive mantle-derived melt and continental crust with high ^87^Sr/^86^Sr ratios (>0.714) and δ^18^O values of 11.8‰ to 19.5‰ (Fig. 5), which is not unreasonable for felsic crust in the whole Central Andean region given that e.g. Damm *et al*.^33^ reported δ^18^O values up to 15.2‰ for Precambrian basement rocks from northern Argentina. The isotope modelling so far assumes simple mixing, which probably approximates behaviour in a deep crustal hot zone, but models involving AFC would likely require greater overall assimilation for the same result, because high-δ^18^O material is removed in the cumulates. Notably, the steady increase in SiO_2_ with no change in δ^18^O value at ca. 7.0‰ (Fig. 5) is consistent with parental magmas that underwent closed-system fractional crystallisation after an initial stage of crustal assimilation by mantle-derived magmas.

We propose, therefore, a model of magmatic evolution for the Altiplano-Puna Volcanic Complex where mantle-derived (primitive) magmas are injected into the felsic continental crust. Upon stagnation, these mantle-derived magmas assimilated basement rocks with high-δ^18^O values and highly radiogenic Sr isotope ratios to form a parental magma with a δ^18^O value of ca. 7.0‰ (Fig. 5). Building on the model of González-Maurel *et al*.^12^ for the western boundary of the Altiplano-Puna Volcanic Complex, parental melts ascended to mid to upper crustal storage levels, where they stalled, differentiated and fractionated^13^, avoiding significant further contamination by e.g. felsic melts derived from the APMB as these mafic melts by-passed the molten APMB body. At these crustal levels, olivine and subsequent clinopyroxene crystallisation occurred, which is consistent with recent thermobarometric estimations performed in Quaternary lavas from the southwestern border of the Altiplano-Puna Volcanic Complex^28^.

In conclusion, volcanic rocks from the most mafic volcanoes at the western border of the Altiplano-Puna Volcanic Complex of the Central Andes have the lowest reported δ^18^O values of 5.7 to 6.2‰ (average = 6.0‰, n = 6) for olivine, whereas clinopyroxene yielded higher δ^18^O values of 6.3 to 7.2‰ (average = 6.7‰, n = 12). These mineral data are consistent with crystallisation from a magma of the same O-isotope composition, allowing a robust δ^18^O estimate of 7.0‰ for the sub-arc, parental magma of the APMB and associated volcanic centres in the Altiplano-Puna Volcanic Complex. This composition may be representative of parental magmas in the wider Central Andean region.

## Methods

### Sample selection and preparation

In this study we analysed crystals from the least silicic materials identified at La Poruña, San Pedro, Paniri, La Poruñita, Palpana and Chela volcanoes. These volcanoes have among the least evolved baseline Sr and Nd isotopic compositions thus far reported for the western boundary of the Altiplano-Puna Volcanic Complex province^12^. Pristine inclusion-free olivine and pyroxene crystals were hand-picked under a binocular microscope.

### Oxygen isotope analysis by laser fluorination

Olivine and pyroxene grains visibly free of alteration or inclusions were selected by hand-picking under a binocular microscope. Laser fluorination (LF) analyses were then carried out at the Department of Geological Sciences, University of Cape Town (UCT), South Africa. The oxygen isotope results are reported in standard δ-notation relative to V-SMOW (Vienna Standard Mean Ocean Water), where δ = [(^18^O/^16^O)_sample_/(^18^O/^16^O)V-SMOW − 1]* 1000. Full analytical details of the laser fluorination method employed at UCT are given in Harris and Vogeli^35^. Measured values of the UCT in-house standard MON GT (Monastery garnet, δ^18^O = 5.38‰) were used to normalise the raw data and correct for drift in the reference gas. The δ^18^O value of MON GT was established by cross-calibration with the UWG-2 garnet standard of Valley *et al*.^36^ and San Carlos olivine. The long-term average difference in δ^18^O values of duplicates of MON GT is 0.15‰, which corresponds to a 2σ S.D. value of 0.15‰. Laser fluorination data are given in Table 1. All analyses gave gas pressures of O_2_ that were consistent with ~100% conversion of mineral to O_2._

## Supplementary information

Supplementary Information.

## Data Availability

The authors declare that all relevant data are available within the article and its supplementary information files.

## References

[1] Davidson JP, Hora JM, Garrison JM, Dungan MA (2005). Crustal forensics in arc magmas. Journal of Volcanology and Geothermal Research.

[2] Beck SL (1996). Crustal-thickness variations in the central Andes. Geology.

[3] Taylor HP (1968). The oxygen isotope geochemistry of igneous rocks. Contributions to mineralogy and Petrology.

[4] Bindeman IN (2005). Oxygen isotope evidence for slab melting in modern and ancient subduction zones. Earth and Planetary Science Letters.

[5] Dallai L, Bianchini G, Avanzinelli R, Natali C, Conticelli S (2019). Heavy oxygen recycled into the lithospheric mantle. Scientific Reports.

[6] Entenmann, J. Magmatic evolution of the Nevados de Payachata complex and the petrogenesis of basaltic andesites in the Central Volcanic Zone of northern Chile. Dissertation, Ph.D. Thesis, Johannes Gutenberg-Universität Mainz, Germany, (1994).

[7] Kay SM, Coira B, Wörner G, Kay RW, Singer BS (2011). Geochemical, isotopic and single crystal 40Ar/39Ar age constraints on the evolution of the Cerro Galan ignimbrites. Bulletin of Volcanology.

[8] Godoy, B. Evolución petrológica de la Cadena Volcánica San Pedro-Linzor (21°30′S–22°10′S), norte de Chile, y su relación con la geodinámica Andina. Dissertation, Ph.D. Thesis, Universidad Católica del Norte, Chile., (2014)

[9] Chang, Y-H. O-Isotopes as tracer for assimilation processes in different magmatic regimes eds. El Misti, S. Peru, E. M. S. & Tapaaca, N. Chile, T. N. C. Dissertation, Diploma Thesis, Georg-August-Universität Göttingen, Germany (2007).

[10] Freymuth H, Brandmeier M, Wörner G (2015). The origin and crust/mantle mass balance of Central Andean ignimbrite magmatism constrained by oxygen and strontium isotopes and erupted volumes. Contributions to Mineralogy and Petrology.

[11] Mattey D, Lowry D, Macpherson C (1994). Oxygen isotope composition of mantle peridotite. Earth and Planetary Science Letters.

[12] González-Maurel O (2019). The great escape: Petrogenesis of low-silica volcanism of Pliocene to Quaternary age associated with the Altiplano-Puna Volcanic Complex of northern Chile (21°10′–22°50′S). Lithos.

[13] Godoy B (2019). Linking the mafic volcanism with the magmatic stages during the last 1 Ma in the main volcanic arc of the Altiplano-Puna Volcanic Complex (Central Andes). Journal of South American Earth Sciences.

[14] de Silva SL (1989). Altiplano-Puna volcanic complex of the central Andes. Geology.

[15] Chmielowski J, Zandt G, Haberland C (1999). The central Andean Altiplano-Puna magma body. Geophysical Research. Letters.

[16] Ward KM, Zandt G, Beck SL, Christensen DH, McFarlin H (2014). Seismic imaging of the magmatic underpinnings beneath the Altiplano-Puna volcanic complex from the joint inversion of surface wave dispersion and receiver functions. Earth and Planetary Science Letters.

[17] Comeau MJ, Unsworth MJ, Ticona F, Sunagua M (2015). Magnetotelluric images of magma distribution beneath Volcán Uturuncu. Bolivia: Implications for magma dynamics. Geology.

[18] Araya Vargas, J. *et al*. Fluid distribution in the Central Andes subduction zone imaged with magnetotellurics. *Journal of Geophysical Research: Solid Earth*, **124**. 10.1029/2018JB016933 (2019).

[19] Zandt G, Leidig M, Chmielowski J, Baumont D, Yuan X (2003). Seismic detection and characterization of the Altiplano-Puna magma body, Central Andes. Pure and Applied Geophysics.

[20] Godoy B (2017). Sr-and Nd-isotope variations along the Pleistocene San Pedro–Linzor volcanic chain, N. Chile: Tracking the influence of the upper crustal Altiplano-Puna Magma Body. Journal of Volcanology and Geothermal Research.

[21] Sellés, D. & Gardeweg, M. Geología del área Ascotán-Cerro Inacaliri, Región de Antofagasta. Servicio Nacional de Geología y Minería, Carta Geológica de Chile, Serie Geología Básica 190:73p., 1 mapa escala 1:100.000. Santiago, Chile (2017).

[22] González-Maurel, O. *et al*. Magmatic differentiation at La Poruña scoria cone, Central Andes, northern Chile: Evidence for assimilation during turbulent ascent processes, and genetic links with mafic eruptions at adjacent San Pedro volcano. *Lithos*, **338–339**, 128–140. https://doi.org/10.1016/j.lithos.2019.03.033 (2019).

[23] Godoy B (2018). Geological evolution of Paniri volcano, Central Andes, northern Chile. Journal of South American Earth Sciences.

[24] Wörner G, Hammerschmidt K, Henjes-Kunst F, Lezaun J, Wilke H (2000). Geochronology (^40^Ar/^39^Ar, K-Ar and He-exposure ages) of Cenozoic magmatic rocks from northern Chile (18-22°S): Implications for magmatism and tectonic evolution of the central Andes. Revista. Geologica de Chile.

[25] Eiler J, Stolper EM, McCanta MC (2011). Intra-and intercrystalline oxygen isotope variations in minerals from basalts and peridotites. Journal of Petrology.

[26] Deegan FM (2016). Pyroxene standards for SIMS oxygen isotope analysis and their application to Merapi volcano, Sunda arc, Indonesia. Chemical Geology.

[27] Risse A, Trumbull RB, Kay SM, Coira B, Romer RL (2013). Multi-stage evolution of late Neogene mantle-derived magmas from the central Andes back-arc in the Southern Puna Plateau of Argentina. Journal of Petrology.

[28] Burns DH, de Silva SL, Tepley FJ, Schmitt AK (2020). Chasing the mantle: Deciphering cryptic mantle signals through Earth’s thickest continental magmatic arc. Earth and Planetary Science Letters.

[29] Bindeman IN, Ponomareva VV, Bailey JC, Valley JW (2004). Volcanic arc of Kamchatka: a province with high-δ^18^O magma sources and large-scale ^18^O/^16^O depletion of the upper crust. Geochimica et Cosmochimica Acta.

[30] Eiler JM (2001). Oxygen isotope variations of basaltic lavas and upper mantle rocks. Reviews in mineralogy and geochemistry.

[31] Eiler JM, Schiano P, Kitchen N, Stolper EM (2000). Oxygen-isotope evidence for recycled crust in the sources of mid-ocean-ridge basalts. Nature.

[32] Jacques G (2014). Geochemical variations in the Central Southern Volcanic Zone, Chile (38–43 S): the role of fluids in generating arc magmas. Chemical Geology.

[33] Damm K.-W. *et al*. Pre-Mesozoic Evolution of the Central Andes; The basement revisited. In Kay, S. M. & Rapela, C. W., eds., *Plutonism from Antarctica to Alaska. Geological Society of America Special Paper* **241**:101–126 (1990).

[34] Ito E, White WM, Göpel C (1987). The O, Sr, Nd and Pb isotope geochemistry of MORB. Chemical Geology.

[35] Harris C, Vogeli J (2010). Oxygen isotope composition of garnet in the Peninsula Granite, Cape Granite Suite, South Africa: constraints on melting and emplacement mechanisms. South African Journal of Geology.

[36] Valley JW, Kitchen N, Kohn MJ, Niendorf CR, Spicuzza MJ (1995). UWG-2, a garnet standard for oxygen isotope ratios: strategies for high precision and accuracy with laser heating. Geochimica et Cosmochimica Acta.

[37] Ryan, W. B. *et al*. Global multi-resolution topography synthesis. *Geochemistry, Geophysics, Geosystems*, **10**(3), 10.1029/2008GC002332 (2009).

