# Supplementary material for: Constraining the sub-arc, parental magma composition for the giant Altiplano-Puna Volcanic Complex, northern Chile
Source: Sci Rep. 2020 Apr 22;10:6864. doi: 10.1038/s41598-020-63454-1 (PMC7176692; doi:10.1038/s41598-020-63454-1)
Supplement: Supplementary file 1 — Supplementary Information. [file 41598_2020_63454_MOESM1_ESM.pdf]

Constraining the sub-arc, parental magma composition for the giant Altiplano-Puna Volcanic Complex, northern Chile

Osvaldo González-Maurel[a,b*], Frances M. Deegan[c], Petrus le Roux[a], Chris Harris[a], Valentin R. Troll[c,d], Benigno Godoy[e]

[a]Department of Geological Sciences, University of Cape Town, South Africa
[b]Departamento de Ciencias Geológicas, Universidad Católica del Norte, Chile
[c]Department of Earth Sciences, Natural Resources and Sustainable Development, Uppsala University, Sweden
[d]Instituto de Estudios Ambientales y Recursos Naturales (i-UNAT), Departamento de Física (Geología), Universidad de Las Palmas de Gran Canaria, Spain
[e]Centro de Excelencia en Geotermia de los Andes (CEGA) y Departamento de Geología, Facultad de Ciencias Físicas y Matemáticas, Universidad de Chile, Chile

*Corresponding author e-mail: gnzosv001@myuct.ac.za; ogm004@alumnos.ucn.cl

Supplementary Information

Oxygen isotope data for Central Andes

Supplementary Table S1: Published mineral oxygen isotope data from Central Andean volcanoes obtained by SIMS, and conventional and laser fluorination of monomineralic crystal separates.

Phase	Method	Number of analysis	$\delta^{18}O$‰ range	$\delta^{18}O$‰ average	Reference
Olivine	Conventional fluorination	5	**5.7 - 6.1**	**5.9**	Entenmann (1994)
	Laser fluorination	14	**5.0 - 8.3**	**6.9**	Godoy (2014); Kay et al. (2011)
Pyroxene	Conventional fluorination	4	**6.8 - 7.2**	**7.1**	Entenmann (1994)
	Laser fluorination	35	**5.5 - 8.7**	**6.1**	Godoy (2014); Chang (2007); Freymuth et al. (2015)
Amphibole	Laser fluorination	28	**5.1 - 6.3**	**5.8**	Chang (2007); Freymuth et al. (2015)
Biotite	Conventional fluorination	1	**6.7**	**6.7**	Entenmann (1994)
Plagioclase	Conventional fluorination	4	**7.4 - 8.3**	**8.0**	Entenmann (1994)
	Laser fluorination	56	**6.5 - 8.1**	**7.4**	Chang (2007); Freymuth et al. (2015); Feeley and Sharp (1995)
Quartz	Conventional fluorination	5	**8.4 - 9.7**	**9.0**	Entenmann (1994)
	Laser fluorination	41	**7.8 - 14.6**	**9.1**	Kay et al. (2010; 2011); Chang (2007); Grocke et al. (2017); Lister (2019)
Zircon	SIMS	78	**5.9 - 9.7**	**6.5**	Kaiser (2014)
Magnetite	Conventional fluorination	4	**2.3 - 5.9**	**4.7**	Entenmann (1994)
	Laser fluorination	35	**2.2 - 4.8**	**3.8**	Chang (2007); Freymuth et al. (2015)
Groundmass	Conventional fluorination	7	**7.1 - 7.6**	**7.4**	Entenmann (1994)
Whole-rock	Conventional fluorination	93	**6.7 - 14.0**	**8.3**	Entenmann (1994); Davidson et al. (1990); Francis et al. (1989); Harmon et al. (1984); Longstaffe et al. (1983)
	Laser fluorination	15	**7.1 - 10.6**	**8.6**	Chang (2007); Freymuth et al. (2015); Feeley and Sharp (1995); Kay et al. (2010)

Supplementary Table S2: Published oxygen isotope data in olivine from Central Andean volcanoes obtained by conventional and laser fluorination of monomineralic crystal separates.

Volcano	Sample	Rock type	$\delta^{18}O$‰	Reference
Conventional fluorination				
Parinacota	PAR-011	Basaltic andesite	5.9	Entenmann (1994)
	PAR-201	Andesite	5.7	Entenmann (1994)
	PAR-220	Mafic andesite	6.1	Entenmann (1994)
	PAR-223	Mafic andesite	6.1	Entenmann (1994)
	PAR-86	Andesite	5.9	Entenmann (1994)
Laser fluorination				
San Pedro	BG-SPL-004	Andesite	6.1	Godoy (2014)
	BG-SPL-010	Basaltic andesite	5.0	Godoy (2014)
Paniri	PANI-10-15	Andesite	6.9	Godoy (2014)
	BG-SPL-022	Basaltic andesite	7.1	Godoy (2014)
Cerro del León	BG-SPL-040	Andesite	6.5	Godoy (2014)
	LEO-10-01	Andesite	8.0	Godoy (2014)
	LEO-10-02	Andesite	7.0	Godoy (2014)
	LEO-10-07	Andesite	7.8	Godoy (2014)
Toconce	TOC-10-02	Andesite	8.3	Godoy (2014)
	TOC-10-04	Andesite	8.2	Godoy (2014)
	TOC-10-08	Dacite	6.3	Godoy (2014)
Cerro Galán	SAF32	Mafic lava	6.4	Kay et al. (2011)
	SAF24	Mafic lava	6.8	Kay et al. (2011)
	SAF76	Mafic lava	6.3	Kay et al. (2011)

Supplementary Table S3: Published oxygen isotope data in pyroxene from Central Andean volcanoes obtained by conventional and laser fluorination of monomineralic crystal separates.

Volcano	Sample	Rock type	δ^{18}O‰	Reference
Conventional fluorination				
Parinacota	PAR-011	Basaltic andesite	7.1	Entenmann (1994)
	PAR-201	Andesite	7.2	Entenmann (1994)
	PAR-223	Mafic andesite	7.2	Entenmann (1994)
	PAR-86	Andesite	6.8	Entenmann (1994)
Laser fluorination				
El Misti	MIS-02-02 PAS	Andesite	6.0	Chang (2007)
	MIS-02-05	Andesite	6.4	Chang (2007)
	MIS-02-06	Andesite	6.0	Chang (2007)
	MIS-02-10 a	Andesite	6.5	Chang (2007)
	MIS-02-107	Andesite	5.8	Chang (2007)
	MIS-02-109 plug	Andesite	6.1	Chang (2007)
	MIS-02-116	Andesite	6.2	Chang (2007)
	MIS-02-118	Andesite	5.7	Chang (2007)
	MIS-02-12	Andesite	6.8	Chang (2007)
	MIS-02-13	Andesite	6.1	Chang (2007)
	MIS-99-10A	Andesite	5.8	Chang (2007)
	MIS-99-10B	Andesite	6.5	Chang (2007)
Taapacá	TAP 97-29/1	Mafic inclusion	6.5	Chang (2007)
	TAP-87-002	Dacite	5.9	Chang (2007)
	TAP-97-06	Andesite	5.8	Chang (2007)
	TAP-97-22	Andesite	5.6	Chang (2007)
	TAP-97-28	Dacite	5.5	Chang (2007)
	TAP-97-29	Dacite	5.6	Chang (2007)
	TAP-97-34	Dacite	6.1	Chang (2007)
Cerro del León	LEO-10-01	Andesite	6.5	Godoy (2014)
Toconce	TOC-10-04	Andesite	8.7	Godoy (2014)
Lascar	LAS 07-01	Andesite	6.0	Freymuth et al. (2015)
	LAS 07-02	Andesite	6.2	Freymuth et al. (2015)
	LAS 07-05	Andesite	6.0	Freymuth et al. (2015)
	LAS 07-07	Andesite	6.1	Freymuth et al. (2015)
	LAS 07-08	Andesite	6.2	Freymuth et al. (2015)
	LAS 07-12	Andesite	5.8	Freymuth et al. (2015)
	LAS 07-16	Andesite	6.0	Freymuth et al. (2015)
	LAS 07-18A	Andesite	6.0	Freymuth et al. (2015)
	LAS 07-18B	Andesite	5.9	Freymuth et al. (2015)
	LAS 07-20	Andesite	6.2	Freymuth et al. (2015)
	LAS 07-22	Andesite	6.1	Freymuth et al. (2015)
	LAS 07-23	Andesite	5.7	Freymuth et al. (2015)
	LAS 07-26	Andesite	5.8	Freymuth et al. (2015)
	LAS 07-28	Andesite	6.0	Freymuth et al. (2015)

AFC-based calculation model

In order to constrain the amount of assimilated crust of all studied samples the AFC-based calculation model of Aitcheson and Forrest (1994) was used, which has been successfully applied in recent petrological studies within the Altiplano-Puna Volcanic Complex (e.g. Godoy et al., 2017; Taussi et al., 2019). In the present model, the local basaltic-andesite end-member is represented by sample CHE-03 from Chela volcano as it shows the lowest $^{87}Sr/^{86}Sr$ (0.705541) and highest $^{143}Nd/^{144}Nd$ (0.512513) ratios of the sample set in González-Maurel et al. (2019). Paleozoic felsic gneisses of the basement exposed in the northern Chilean Precordillera at 21°04'S to 22°01'S were selected as crustal contaminants (SiO_2 = 54.0 to 69.3 wt%, MgO = 1.6 to 7.1 wt%, Sr = 114 to 1091 ppm, and $^{87}Sr/^{86}Sr$ = 0.7085 to 0.7278; Lucassen et al., 2001). Note that the sample with the highest Sr isotope ratio, and associated Sr concentration, is used as the contaminant for the AFC model (Supplementary Table S4). The mineral assemblage is estimated based on the petrographic analysis in González-Maurel et al. (2019). Models were generated separately for the Pliocene (Chela and Palpana) and Quaternary (La Poruña, San Pedro, Paniri and La Poruñita) mafic magmas as they show slightly different mineral proportions (Supplementary Table S4). An AFC-based calculation model was used to constrain the amount of assimilated crust of the studied rocks using Eq. (5) from the RAFT model by Aitcheson and Forrest (1994) (Supplementary Table S5). Results of RAFT-modelling indicate the degree of crustal assimilation (excluding CHE-03) to be ca. 12% for Palpana, 24 to 28% for La Poruña, 22% for San Pedro, 19% for Paniri and 26% for La Poruñita.

Supplementary Table S4: AFC model parameters (*after* DePaolo, 1981) for erupted Pliocene and Quaternary magmas at 21°10'-22°25'S. Bulk D according mineral/melt partition coefficients for basaltic and basaltic-andesite liquids from Rollinson (1993).

	**Initial magma composition**	**Crustal contaminant**
Location	Chela	Basement gneiss
Reference	González-Maurel et al. (2019)	Lucassen et al. (2001)
Sample	CHE-03	4/316
Major oxides and trace elements		
SiO_2 (wt%)*	55.50	67.01
Al_2O_3 (wt%)*	18.20	15.52
FeO_t (wt%)*	7.31	8.26
MgO (wt%)*	3.92	2.20
CaO (wt%)*	7.47	1.83
Na_2O (wt%)*	4.04	2.53
K_2O (wt%)*	1.44	3.28
Sr (ppm)	793	185
Nd (ppm)	18	37
Radiogenic isotopes		
$^{87}Sr/^{86}Sr$	0.705541	0.727770
$^{143}Nd/^{144}Nd$	0.512513	0.512087
	**Pliocene**	**Quaternary**
Mineral assemblage		
Plagioclase (vol%)	70	60
Olivine (vol%)	5	10
Orthopyroxene (vol%)	10	15
Clinopyroxene (vol%)	15	15
Bulk partition coefficient		
D_{Sr}	1.29	1.11
Conditions		
r = Ma / Mc	0.5	0.5

$_t$ = total Fe as Fe^{2+}

*Recalculated to 100% anhydrous

Supplementary Table S5: Calculated amounts of crustal assimilation for Pliocene and Quaternary mafic magmas using Eq. (5) (Aitcheson and Forrest, 1994). Estimated remaining melt fraction (F) from AFC modelling in this work (Supplementary Table S4).

F (%)	Pliocene magma		Quaternary magma		Assimilated crust (%)
	Sr (ppm)	$^{87}Sr/^{86}Sr$	Sr (ppm)	$^{87}Sr/^{86}Sr$	
100	793	0.705541	793	0.705541	0
95	740	0.705815	754	0.705812	5
90	689	0.706120	716	0.706109	9
85	640	0.706462	678	0.706436	13
80	592	0.706847	640	0.706796	17
75	546	0.707282	603	0.707195	20
70	502	0.707776	567	0.707640	23
65	459	0.708339	531	0.708137	26
60	419	0.708984	496	0.708696	29
55	380	0.709728	461	0.709328	31
50	343	0.710589	427	0.710047	33

O-isotope modelling

Supplementary Table S6: Mixing components for the end-member compositions of hypothetical mantle (M) (SiO_2 = 51 wt%; Sr = 113 ppm; $^{87}Sr/^{86}Sr$ = 0.703; $\delta^{18}O$ = 5.7‰; *after* Harmon et al., 1981; Ito et al., 1987; Hoffmann, 1988; Davidson et al., 1990; Harmon and Hoefs, 1995).

Component	SiO_2 (wt%)	Sr (ppm)	$^{87}Sr/^{86}Sr$	$\delta^{18}O$	Reference
C1	67	185	0.72777	11.8	Damm et al. (1990); Lucassen et al. (2001)
C2	70	200	0.71400	19.5	Davidson et al. (1990)
Sediments	70	300	0.71000	25	Eiler et al. (1997)

Supplementary references

1. Entenmann, J., 1994. Magmatic evolution of the Nevados de Payachata complex and the petrogenesis of basaltic andesites in the Central Volcanic Zone of northern Chile. Dissertation, Ph.D. Thesis, Johannes Gutenberg-Universität Mainz, Germany.
2. Godoy, B., 2014. Evolución petrológica de la Cadena Volcánica San Pedro-Linzor (21º30'S-22º10'S), norte de Chile, y su relación con la geodinámica Andina. Dissertation, Ph.D. Thesis, Universidad Católica del Norte, Chile.
3. Kay, S.M., Coira, B., Wörner, G., Kay, R.W., Singer, B.S., 2011. Geochemical, isotopic and single crystal $^{40}Ar/^{39}Ar$ age constraints on the evolution of the Cerro Galan ignimbrites. Bulletin of Volcanology, 73(10):1487-1511. https://doi.org/10.1007/s00445-010-0410-7.
4. Chang, Y-H., 2007. O-Isotopes as tracer for assimilation processes in different magmatic regimes (El Misti, S. Peru and Tapaaca, N. Chile). Dissertation, Diploma Thesis, Georg-August-Universität Göttingen, Germany.
5. Freymuth, H., Brandmeier, M., Wörner, G., 2015. The origin and crust/mantle mass balance of Central Andean ignimbrite magmatism constrained by oxygen and strontium isotopes and erupted volumes. Contributions to Mineralogy and Petrology, 169(6):58. https://doi.org/10.1007/s00410-015-1152-5.
6. Feeley, T.C., Sharp, Z.D., 1995. $^{18}O/^{16}O$ isotope geochemistry of silicic lava flows erupted from Volcán Ollagüe, Andean Central Volcanic Zone. Earth and Planetary Science Letters, 133(3-4):239-254. https://doi.org/10.1016/0012-821X(95)00094-S.
7. Kay, S.M., Coira, B.L., Caffe, P.J., Chen, C.H., 2010. Regional chemical diversity, crustal and mantle sources and evolution of central Andean Puna plateau ignimbrites. Journal of Volcanology and Geothermal Research, 198(1-2):81-111. https://doi.org/10.1016/j.jvolgeores.2010.08.013.
8. Grocke, S.B., de Silva, S.L., Iriarte, R., Lindsay, J.M., Cottrell, E., 2017. Catastrophic caldera-forming (CCF) monotonous silicic magma reservoirs: Geochemical and petrological constraints on heterogeneity, magma dynamics, and eruption dynamics of the 3.49 Ma Tara Supereruption, Guacha II Caldera, SW Bolivia. Journal of Petrology, 58(2):227-260. https://doi.org/10.1093/petrology/egx012.
9. Lister, J., 2019. Petrogenesis of lavas from Volcano Azufre, Northern Chile: evidence for crustal input. Dissertation, M.Sc. Thesis, University of Cape Town, South Africa.
10. Kaiser, J.F., 2014. Understanding Large Resurgent Calderas and Associated Magma Systems: The Pastos Grandes Caldera Complex, Southwest Bolivia. Dissertation, Ph.D. Thesis, Oregon State University, U.S.A.
11. Davidson, J.P., McMillan, N.J., Moorbath, S., Wörner, G., Harmon, R.S., Lopez-Escobar, L., 1990. The Nevados de Payachata volcanic region (18 S/69 W, N. Chile) II. Evidence for widespread crustal involvement in Andean magmatism. Contributions to Mineralogy and Petrology, 105(4):412-432. https://doi.org/10.1007/BF00286829.
12. Francis, P.W., Sparks, R.S.J., Hawkesworth, C.J., Thorpe, R.S., Pyle, D.M., Tait, S.R., Mantovani, M.S., McDermott, F., 1989. Petrology and geochemistry of volcanic rocks of the Cerro Galan caldera, northwest Argentina. Geological Magazine, 126(5):515-547. https://doi.org/10.1017/S0016756800022834.
13. Harmon, R.S., Barreiro, B.A., Moorbath, S., Hoefs, J., Francis, P.W., Thorpe, R.S., Deruelle, B., McHugh, J., Viglino, J.A., 1984. Regional O-, Sr-, and Pb-isotope relationships

in late Cenozoic calc-alkaline lavas of the Andean Cordillera. Journal of the Geological Society, 141(5):803-822. https://doi.org/10.1144/gsjgs.141.5.0803.

14. Longstaffe, F.J., Clark, A.H., McNutt, R.H., Zentilli, M., 1983. Oxygen isotopic compositions of Central Andean plutonic and volcanic rocks, latitudes 26–29 south. Earth and Planetary Science Letters, 64(1):9-18. https://doi.org/10.1016/0012-821X(83)90048-1.

15. Aitcheson, S.J., Forrest, A.H., 1994. Quantification of crustal contamination in open magmatic systems. Journal of Petrology, 35(2):461-488. https://doi.org/10.1093/petrology/35.2.461.

16. Godoy, B., Wörner, G., Le Roux, P., de Silva, S., Parada, M.Á., Kojima, S., González-Maurel, O., Morata, D., Polanco, E., Martínez, P., 2017. Sr-and Nd-isotope variations along the Pleistocene San Pedro–Linzor volcanic chain, N. Chile: Tracking the influence of the upper crustal Altiplano-Puna Magma Body. Journal of Volcanology and Geothermal Research, 341:172-186. https://doi.org/10.1016/j.jvolgeores.2017.05.030.

17. Taussi, M., Godoy, B., Piscaglia, F., Morata, D., Agostini, S., Le Roux, P., González-Maurel, O., Gallmeyer, G., Menzies, A., Renzulli, A., 2019. The upper crustal magma plumbing system of the Pleistocene Apacheta-Aguilucho Volcanic Complex area (Altiplano-Puna, northern Chile) as inferred from the erupted lavas and their enclaves. Journal of Volcanology and Geothermal Research, 373:179-198. https://doi.org/10.1016/j.jvolgeores.2019.01.021.

18. González-Maurel, O., le Roux, P., Godoy, B., Troll, V.R., Deegan, F.M., Menzies, A., 2019. The great escape: Petrogenesis of low-silica volcanism of Pliocene to Quaternary age associated with the Altiplano-Puna Volcanic Complex of northern Chile (21° 10′-22° 50′ S). Lithos, 346-347:105162. https://doi.org/10.1016/j.lithos.2019.105162.

19. Lucassen, F., Becchio, R., Harmon, R., Kasemann, S., Franz, G., Trumbull, R., Wilke, H.G., Romer, R.L., Dulski, P., 2001. Composition and density model of the continental crust at an active continental margin—the Central Andes between 21 and 27 S. Tectonophysics, 341(1-4):195-223. https://doi.org/10.1016/S0040-1951(01)00188-3.

20. DePaolo, D.J., 1981. Trace element and isotopic effects of combined wallrock assimilation and fractional crystallization. Earth and planetary science letters, 53(2):189-202. https://doi.org/10.1016/0012-821X(81)90153-9.

21. Rollinson, H.R., 1993. Using Geochemical Data: Evaluation, Presentation, Interpretation. Longman Scientific and Technical (352 pp.).

22. Harmon, R.S., Thorpe, R.S., Francis, P.W., 1981. Petrogenesis of Andean andesites from combined O–Sr isotope relationships. Nature, 290(5805):396-399. https://doi.org/10.1038/290396a0.

23. Ito, E., White, W.M., Göpel, C., 1987. The O, Sr, Nd and Pb isotope geochemistry of MORB. Chemical Geology, 62(3-4):157-176. https://doi.org/10.1016/0009-2541(87)90083-0.

24. Hofmann, A.W., 1988. Chemical differentiation of the Earth: the relationship between mantle, continental crust, and oceanic crust. Earth and Planetary Science Letters, 90(3):297-314. https://doi.org/10.1016/0012-821X(88)90132-X.

25. Davidson, J.P., McMillan, N.J., Moorbath, S., Wörner, G., Harmon, R.S., Lopez-Escobar, L., 1990. The Nevados de Payachata volcanic region (18°S/69°W, N. Chile) II.

Evidence for widespread crustal involvement in Andean magmatism. Contributions to Mineralogy and Petrology, 105(4):412-432. https://doi.org/10.1007/BF00286829.

26. Harmon, R.S., Hoefs, J., 1995. Oxygen isotope heterogeneity of the mantle deduced from global ^{18}O systematics of basalts from different geotectonic settings. Contributions to Mineralogy and Petrology, 120(1):95-114. https://doi.org/10.1007/BF00311010.

27. Damm K.-W., Pichowiak S., Harmon R. S., Todt W., Omarini R., Niemeyer H., 1990. Pre-Mesozoic Evolution of the Central Andes; The basement revisited. in Kay, S.M., Rapela, C. W., eds., Plutonism from Antarctica to Alaska. Geological Society of America Special Paper 241:101-126.

28. Eiler, J.M., Farley, K.A., Valley, J.W., Hauri, E., Craig, H., Hart, S.R., Stolper, E.M., 1997. Oxygen isotope variations in ocean island basalt phenocrysts. Geochimica et Cosmochimica Acta, 61(11):2281-2293. https://doi.org/10.1016/S0016-7037(97)00075-6.
